# A Systematic Review of Estrogens as Emerging Contaminants in Water: A Global Overview Study from the One Health Perspective

**DOI:** 10.3390/jox15050148

**Published:** 2025-09-13

**Authors:** Rhitor Lorca da Silva, Marco Antonio Lima e Silva, Tiago Porfírio Teixeira, Thaís Soares Farnesi de Assunção, Paula Pinheiro Teixeira, Wagner Antonio Tamagno, Thiago Lopes Rocha, Julio Cesar de Souza Inácio Gonçalves, Matheus Marcon

**Affiliations:** 1 Laboratório de Estudos Farmacológicos, Instituto de Ciências Biológicas e Naturais, Universidade Federal do Triângulo Mineiro, Uberaba 38025-015, Brazil; d-20098@uftm.edu.br (R.L.d.S.); d201920242@uftm.edu.br (M.A.L.e.S.); d201920240@uftm.edu.br (T.P.T.); thais.assuncao@uftm.edu.br (T.S.F.d.A.); paulapinheirot@gmail.com (P.P.T.); 2 Laboratório de Zebrafish, Instituto de Ciências Biológicas e Naturais, Universidade Federal do Triângulo Mineiro, Uberaba 38025-015, Brazil; 3 School of Health Sciences, Purdue University, West Lafayette, IN 47906, USA; tamagnowagner.99@gmail.com; 4 Laboratório de Biotecnologia Ambiental e Ecotoxicologia, Instituto de Patologia Tropical e Saúde Pública, Universidade Federal de Goiás, Goiânia 74605-050, Brazil; thiagorochabio20@ufg.br; 5 Laboratório de Processos Ambientais, Instituto de Ciências Tecnológicas e Exatas, Universidade Federal do Triângulo Mineiro, Uberaba 38064-200, Brazil; julio.goncalves@uftm.edu.br

**Keywords:** estradiol, estrone, estriol, ethinylestradiol, estrogenic compounds, endocrine disruptors

## Abstract

The widespread presence of estrogens in aquatic environments represents a One Health concern, as it simultaneously threatens environmental integrity, wildlife health, and human well-being. These compounds, widely used in human and veterinary medicine, are excreted in partially or unmetabolized forms and persist in the environment due to the inefficiency of conventional water treatment systems in removing them. This systematic review provides a global overview of the occurrence of estrogens in water resources. We synthesized data on study characteristics, estrogen compounds detected, their concentrations, types of water bodies, and geographic locations. In total, 39 estrogens, including natural, synthetic, and metabolite forms, were reported at concentrations ranging from 0.002 to 10,380,000.0 ng/L across 40 water body types in 59 countries on all continents. The most frequently detected compounds were estrone, estradiol, and ethinylestradiol. Estrogens were predominantly identified in wastewater treatment plant effluents, rivers, lakes, surface waters, and even drinking water sources. These findings underscore the estrogen contamination and its potential to disrupt endocrine functions across species, posing serious implications for ecosystems. Within the One Health framework, this review highlights the urgent need for integrated strategies to improve water quality monitoring, develop advanced treatment technologies, and update regulatory standards to address the multifaceted risks posed by estrogenic contaminants.

## 1. Introduction

The One Health approach, endorsed by major global health institutions, recognizes the interconnectedness of human, animal, and environmental health. This integrative framework is crucial for addressing contemporary challenges, such as the contamination of water resources by emerging pollutants, which transcend disciplinary boundaries and necessitate coordinated global action [1,2]. Environmental pollutants, including endocrine-disrupting chemicals (EDCs), can simultaneously impact ecosystems, wildlife, and human populations, making them a central concern within the One Health context [3].

In recent decades, the global population has experienced significant growth, accompanied by the expansion of urban, industrial, and agricultural sectors [4]. This growth has increased human impacts on the environment, especially concerning its overexploitation and contamination, which threaten ecosystems and public health [5]. Many contaminants are being introduced daily into various environmental compartments, particularly in the water resources, such as plastics [6], drugs [7], heavy metals [8], pesticides [9], and even radioactive substances [10].

Among these emerging contaminants, estrogens have drawn the attention of the scientific community due to their endocrine-disrupting potential [11]. Endocrine-disrupting substances are exogenous molecules that interfere with the endocrine system and can cause adverse effects on organisms, their offspring, or entire populations [12,13,14]. These substances are known to disrupt hormonal balance by either antagonizing natural endocrine hormones, mimicking their effects, overstimulating receptors, or altering receptor synthesis (such as downregulation or upregulation), leading to a variety of harmful interactions with endogenous hormones [15].

Estrogens are a group of fat-soluble steroid hormones derived from cholesterol, characterized by 18 carbon atoms in a cyclic structure with three cyclohexane rings and a cyclopentyl ring [16]. Endogenous estrogens such as estrone, estradiol, and estriol play vital roles in regulating the female reproductive system and developing secondary sexual characteristics [17]. Synthetic estrogens, including ethinylestradiol, mestranol, and quinestrol, are widely used oral contraceptives and hormone replacement therapies to treat hypoestrogenism, prevent osteoporosis, and alleviate menopausal symptoms [18,19].

The extensive use of estrogens in human and veterinary medicine, associated with their excretion of partially or non-metabolized forms in urine and feces, facilitates their release into the environment. Estrogens enter the ecosystems directly through industrial, urban, and agricultural effluents release or indirectly via effluent discharge from conventional wastewater treatment plants that currently are ineffective at fully removing these compounds [15,20]. Some advanced treatment processes, such as hydrodynamic cavitation [21], Fenton’s approach [22], enzymatic degradation [23], adsorption [24], and ozonation [25], are considered promising alternatives for removing estrogens. However, these advanced treatments have disadvantages such as high maintenance costs, poor selectivity, waste product formation, and high complexity for large-scale application [17]. Additionally, improper disposal of unused estrogens contributes a lesser portion to environmental contamination [5,19,26].

Due to their chemical stability, estrogens resist natural degradation processes, leading to their accumulation in environmental compartments, particularly water bodies [27,28,29,30]. The persistent presence of estrogens in the environment negatively affects local ecosystems and human health, as people may be exposed to contaminated drinking water over long periods [31,32]. Research has linked chronic exposure to estrogens with some disorders, such as homeostasis disruption [32], increased cancer risk [33,34], gestational complications [35], congenital malformations [36], obesity [37], and reduced fertility due to impaired spermatogenesis [38,39]. These health consequences align with the core of the One Health approach, which recognizes the need to address environmental contaminants not only as ecological threats but also as direct and indirect contributors to public and animal health burdens [1].

Previous studies have reported the pollution of aquatic environments with estrogens, such as the studies about the Periyar River, in Kerala (India) [40], European aquatic environment [41], and Yundang Lagoon in China [42], as well as the most comprehensive studies on the global occurrence of estrogens in water resources [43] and drinking water [44]. Additionally, another study discussed the impact of estrogens on the environment and humans [45]. Although some evidence shows the occurrence of estrogens in water resources and drinking water, the literature still lacks studies that compile deeper information on the pollution of water resources worldwide with all estrogens and their metabolites, reporting the geographical occurrence by country and the concentration of each estrogen found in each water body, discussing their implications for health from the One Health framework.

To fill this gap, we conducted this review study to provide a comprehensive overview of the occurrence of estrogens in all water resources worldwide with no restrictions on periods, geographic location, or specific estrogen. We also discuss the impacts on living organisms, current regulatory frameworks, and address the challenges associated with environmental monitoring. These aspects were analyzed through the lens of the One Health framework, which acknowledges the interconnectedness of human, animal, and environmental health and emphasizes the need for integrated efforts to improve public health outcomes. Ultimately, this review aims to provide ecotoxicological insights that support more effective regulations for water and wastewater treatment processes, particularly in terms of estrogen removal.

## 2. Methods

### 2.1. Search Strategy

We conducted this review study in accordance with the PRISMA guidelines [46]. On 10 October 2022, we performed a literature search in Scopus, PubMed, and Web of Science databases using terms related to the estrogens (estradiol; estrone; estriol; ethinylestradiol; mestranol; quinestrol; hydroxyestrones; ethynodiol diacetate; esterified estrogens; conjugated estrogens; catechol estrogens) combined with broad terms that describe the water resources (water resources; drinking water; effluent; surface freshwater; wastewater; residual water; water body; tap water). The complete search strategies employed in each database are available in Supplementary Material S1. This systematic review was registered in Open Science Framework (https://osf.io/rkvtf/) (accessed on 11 September 2024).

### 2.2. Eligibility Screening

After conducting the literature search, the records found in the databases were imported into the Rayyan^®^ web platform [47], which was used throughout the selection process. Initially, duplicates were identified and removed after confirming them by reviewing titles/abstracts. Subsequently, studies were selected based on predefined inclusion/exclusion criteria, starting with title/abstract screening and proceeding to full-text screening. The selection process was carried out by two independent reviewers (R.L.S. and T.P.T.), with any discrepancies resolved by a third reviewer (M.A.L.S.).

Studies were included in this review if they met the following criteria: (1) original studies; (2) reported the occurrence of estrogens in surface water, groundwaters, tap water, drinking water, or effluent from wastewater or sewage treatment plants; (3) reported the occurrence of estrogens in wastewater that was discharged into water resources (e.g., river, lake, sea). Studies were excluded if they met the following criteria: (1) not written in English; (2) full texts were not available; (3) the analyzed sample was enriched with estrogens; (4) sample analysis was performed by bioassay methods or using polar organic chemical integrative samplers; (5) estrogens were not detected in analyzed samples or were below the limit of detection (LOD); (6) estrogen concentration found was unclear; (7) estrogen concentration was expressed in molar unit; (8) the sample was collected within a water treatment plant.

### 2.3. Data Extraction

All extracted data were identified through analysis of the full text, tables, and Supplementary Material of the selected studies. The collected information included: (1) general details (title, first author’s surname, and year of publication); (2) the name of the estrogen detected; (3) the concentration of estrogen detected; (4) the water body in which the estrogen was found; (5) the geographic location of water sampling (e.g., city, state or province, and country, when available). Estrogen concentration data were shown in this review as reported in each study (e.g., minimum-maximum concentration, mean concentration, or concentration below the limit of quantification (LOQ), when available), and to facilitate comparison between concentrations detected in different studies, we showed all concentrations in nanograms per liter (ng/L). All graphs were plotted using the programs Microsoft PowerPoint^®^ and Excel^®^ (version 16.93.1).

## 3. Results

### 3.1. Studies Selected

**Figure 1:**
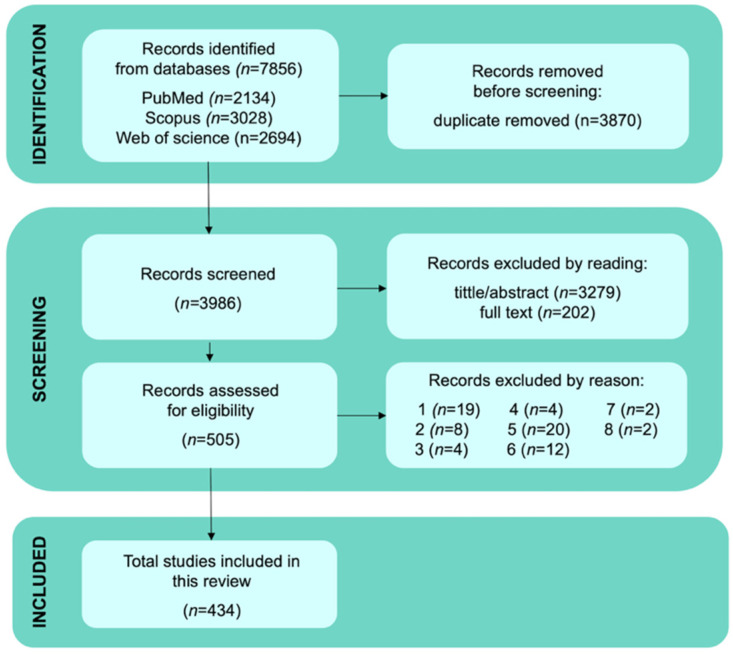
**Flowchart of study screening and selection process.** The diagram details the database searches, the number of abstracts and full texts screened, the records assessed for eligibility, and the studies included in the review. The numbers indicating exclusion reasons can be assessed in full in the methodology section.

We have identified a total of 7856 articles in the selected databases: PubMed (2134), Scopus (3028), and Web of Science (2694). We removed 3870 duplicate records, and 3986 articles were screened. Then, 3279 studies were excluded after titles/abstracts screening, and 202 were excluded after full-text screening because they did not meet the inclusion criteria, leaving 505 studies for eligibility assessment. After analyzing the full text and Supplementary Material of the eligible studies, 71 studies were excluded based on the exclusion criteria. A total of 434 studies were selected, as outlined in Figure 1.

### 3.2. Historical Overview

**Figure 2:**
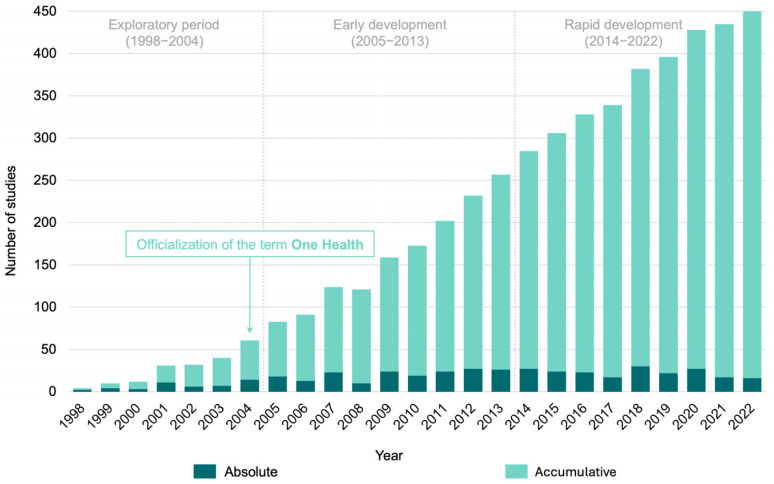
**Studies published concerning the occurrence of estrogens in water resources.** Absolute and cumulative number of studies.

The absolute and cumulative number of studies on estrogens in water resources are shown in Figure 2. All the 434 studies included in this review were published between 1998 and 2022. The first two studies on the presence of estrogens in water resources were published in 1998. One study detected estrone, estradiol, and ethinylestradiol at concentrations ranging from 1.4 to 76 ng/L in the effluent of a wastewater treatment plant, receiving primarily domestic effluent, discharging into British rivers [48]. Another study detected estrone, estriol, and estradiol at concentrations ranging from 6 to 72 ng/L in the effluent of sewage treatment plants in several Canadian cities (Burlington, Dundas, Edmonton, Guelph, Montreal) [49]. These studies have alerted the scientific community that estrogens are environmental contaminants in sewage effluents, indicating their potential impact on human and environmental health. Notably, this period marked the initial recognition of the relevance of emerging contaminants like estrogens within the One Health framework, emphasizing the interconnected risks to human, animal, and ecosystem health posed by anthropogenic pollutants [50].

From 1998 until 2004, research on this topic was exploratory, with few studies published (*n* = 47; 10.82%). The year 2004 had the highest number of publications during this period (*n* = 14; 3.22%), and coincidentally, represents the year in which the creation of the term One Health was made official [51]. As analytical methods for detecting and quantifying estrogens were developed and popularized, and the One Health ideology was growing as an approach necessary to achieving global health [1], the number of studies increased steadily. Between 2005 and 2013, 184 studies were published (42.39%), reflecting a period of early development. This trend continued, with an exponential rise in publications from 2014 and 2022, during which 203 studies were published (46.77%).

### 3.3. Geographical Distribution of Estrogen in Water Resources

**Figure 3:**
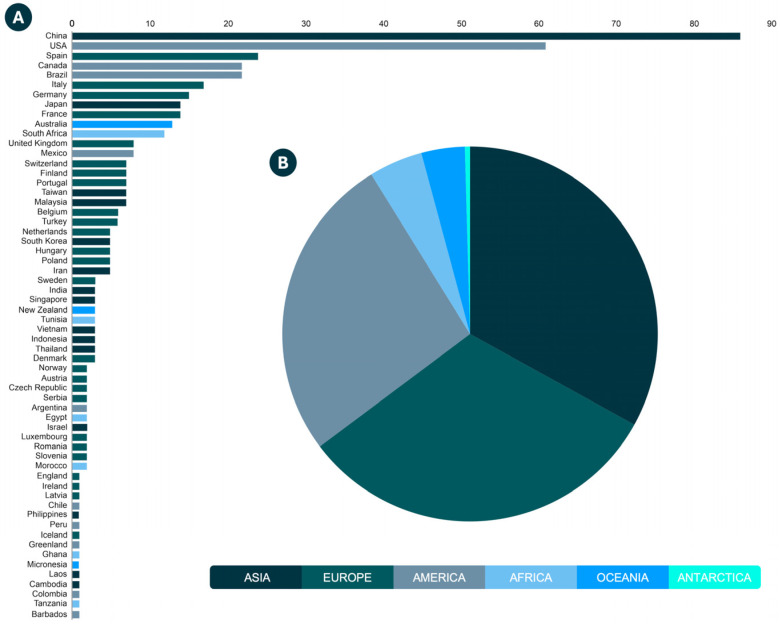
**Occurrence of estrogens in water by country and continent.** The number of studies that reported estrogens in the water resources of each country (**A**) and each continent (**B**).

The results showed the occurrence of estrogens in water resources of 59 countries, mainly of the China (*n* = 86; 19.81%), United States of America (USA) (*n* = 61; 14.05%), Spain (*n* = 24; 5.52%), Canada, Brazil (*n* = 22; 5.06% each), Italy (*n* = 17; 3.91%), Germany (*n* = 15; 3.45%), France, Japan (*n* = 14; 3.22% each), and Australia (*n* = 13; 2.99%) (Figure 3A). The data compiled by continent revealed that most studies were from water resources of Asian countries (*n* = 150; 34.56%), followed by European (*n* = 144; 33.17%), American (*n* = 120; 27.64%), African (*n* = 21; 4.83%), Oceanian (*n* = 17; 3.91%), and Antarctic continent (*n* = 1; 0.23%), highlighting the global extent of water estrogens contamination (Figure 3B).

**Figure 4:**
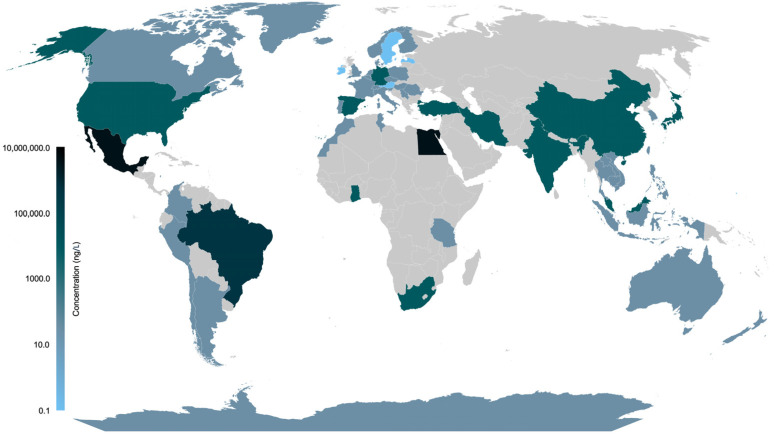
**Overview of the geographical distribution of estrogens in water worldwide.** The maximum concentration (ng/L) found is shown by country. Light and dark colors represented the lowest and highest, respectively.

Furthermore, in 6 studies included in this review, the place of water sampling was unclear. An overview of the geographical distribution of estrogens found in water worldwide, and the maximum concentration reported by country, is shown in Figure 4. All data are available in Supplementary Material S2.

### 3.4. Estrogens Detected in Water Resources

Revised data showed that 39 estrogens have already been detected in a wide range of concentrations (0.002 to 10,380,000.0 ng/L) in water resources worldwide (Table 1). The most common estrogens found in water resource were estrone (*n* = 336; 77.41%), estradiol (17β-estradiol) (*n* = 327; 75.34%), ethinylestradiol (*n* = 216; 49.76%), and estriol (*n* = 171; 39.40%), following by alfatradiol (17α-estradiol) (*n* = 38; 8.75%), estrone-3-sulfate (*n* = 28; 6.45%), estradiol-3-sulfate (*n* = 16; 3.68%), estriol-3-sulfate, mestranol (*n* = 7; 1.61% each), 16α-hydroxyestrone (*n* = 5; 1.15%), ethinylestradiol-3-sulfate, estrone-3-β-D-glucuronide (*n* = 4; 0.92% each), estradiol-17-glucuronide, estrone-3-glucuronide (*n* = 3; 0.69% each), estradiol-17-acetate, estradiol-17-β-D-glucuronide, estradiol-3-glucuronide (*n* = 2; 0.46% each), diClE2, estradiol-glucoside, estradiol-3-β-D-glucuronide, estradiol-17-sulfate, estradiol-17-valerate, estriol-3-glucuronide, estriol-3-β-D-glucuronide, estriol-16-glucuronide, ethinylestradiol-3-glucuronide, E2-diS, E2-monoS, E2-S&G, monoBrEE2, 2-bromo-17β-estradiol, 2-hydroxyestrone, 4-hydroxyestrone, 2,4-dibromo-17β-estradiol, 2,4-dichloro-17β-estradiol, 4-chloro-estriol, 4-chloro-17α-ethynylestradiol, 16-ketoestradiol, and 17α-estradiol-3-sulfate (*n* = 1; 0.23% each). The minimum and maximum concentrations for each estrogen found in water resources are described in Table 1.

The occurrence of estrogens has been described in 40 types of water bodies. Studies were found estrogens mainly in effluent of wastewater treatment plant (*n* = 291; 67.05%), river (*n* = 183; 42.16%), and effluent of sewage treatment plants (*n* = 50; 11.52%), following by lake, surface water (*n* = 30; 6.91% each), drinking water (*n* = 23; 5.29%), canal water, stream (*n* = 13; 2.99% each), lagoon (*n* = 12; 2.76%), creek, well water, seawater (*n* = 11; 2.53% each), groundwater (*n* = 10; 2.30%), tap water (*n* = 9; 2.07%), coastal water, estuarine, reservoir (*n* = 6; 1.38% each), untreated wastewater discharged into surface waters (*n* = 5; 1.15%), pond (*n* = 4; 0.92%), dam water, effluent of industrial wastewater treatment plant, tributary, wetland water (*n* = 3; 0.69% each), effluent of livestock wastewater treatment plant, storm water runoff (*n* = 2; 0.46% each), drainage water, effluent of domestic wastewater treatment plant, effluent of fish farm, embayments, ocean, reclaimed water, residual water, bay water, spring water, treated water, waterway, swimming pool water, and unidentified water (*n* = 1; 0.23% each). The minimum and maximum concentrations in ng/L of the estrogens detected in each water body are described in Table 2. All information regarding study reference, author, estrogen concentration found, and geographic location is available in Supplementary Material S2.

## 4. Discussion

### 4.1. General

Our review identified that 434 studies published between 1998 and 2022 found estrogens in water resources. Revised data revealed an exponential rise in the total number of publications per year on this topic, from just 2 studies in 1998 to 16 in 2022. This notable growth in published research likely reflects advances in measurement techniques and increased awareness and concern among scientists about emerging contaminants. Specifically, estrogens are widely used in medicine and livestock, and these molecules demonstrate high chemical stability, even in environmental conditions [52]. Although the total number of studies has increased exponentially over the past decade, the annual count of articles per year has remained steady (with an average of 22.9 and a peak of 30 studies). This suggests that this research area still needs more focus and discussion within the scientific community.

The occurrence of estrogens in water resources across 59 countries highlights their status as widespread environmental pollutants with global relevance. Water contamination by estrogen crosses geographic, political, and economic borders, as estrogens have been detected in water bodies worldwide, including in both developed and developing nations, as well as in remote and fragile ecosystems, like Erebus Bay in Antarctica. Despite this widespread contamination, there is no clear link between the number of studies conducted and a country’s level of development, indicating significant gaps in monitoring. Notably, most studies included in this review came from China (*n* = 86; 19.81%) and the USA (*n* = 61; 14.05%).

In total, we identified 39 estrogens that have been reported in water resources worldwide. Many of these estrogens are used in pharmaceuticals, which aligns with the most common estrogens in therapeutic practice, such as estradiol (also known as 17β-estradiol, the primary estrogen produced by the ovaries, used in contraceptives and hormone replacement therapy) [53], ethinylestradiol and mestranol (synthetic drugs frequently found in contraceptives) [54,55], and estriol (a metabolite of estradiol used in hormone replacement therapy) [56]. Other metabolites and derivatives excreted in the urine were also found, including estrone (a less potent metabolite of estradiol), ethinylestradiol (an active metabolite of mestranol), estradiol-3-glucuronide and estradiol-17-sulfate, ethinylestradiol-3-glucuronide and ethinylestradiol-3-sulfate, and estriol-3-glucuronide and estriol-3-sulfate. These last are conjugates of active estrogens with glucuronic acid and sulfuric acid, respectively [52,57].

Studies have documented the occurrence of estrogens in many water bodies, primarily in effluents from water treatment plants. This highlights the inefficiency of these plants in removing estrogens from water, as many studies have detected these compounds in their effluents. Even more concerning, estrogens are often found in water sources that are directly accessible to humans, such as rivers, lakes, surface water, and, alarmingly, in drinking water. In these locations, estrogens tend to be present at the highest concentrations. This evidence underscores the extent of water pollution caused by estrogens and draws attention to their potential impact on humans.

Prolonged exposure to relevant environmental concentrations of estrogens induced several damages in living organisms [58,59]. For example, in fish species, which serve as direct aquatic bioindicators, there are changes in population gender structure [60,61], disruption in egg production and quality [62,63], cytogenotoxicity in blood cells [64], alterations in the development of ovaries and ovotestis, and delays in gonadal development retardation [65]. Additionally, the induction of hepatocarcinogenic processes has been observed [66]. While there is no conclusive evidence yet that estrogens in water directly harm humans, this issue requires closer attention due to the hazardous effects reported in animals. Elevated estrogen levels in humans have been associated with polycystic ovary syndrome [67], endometriosis [68], and various cancers, including endometrial, ovarian, breast, gastric, lung, liver, pituitary, and thyroid cancer [69,70,71,72,73,74], as well as hypogonadism and gynecomastia in men [75,76].

Previous studies have also shown the widespread presence of other drugs in water sources, including analgesics [77], antifungals [78], antibacterials [79,80,81], antiparasitics [82], antineoplastics [83,84,85,86], non-steroidal anti-inflammatory drugs [87,88], opioids [89], and psychotropic drugs [90,91,92,93,94]. Pharmaceuticals are increasingly found in water sources [95,96,97,98,99,100], and reports from the World Health Organization (2012) and the Organization for Economic Co-operation and Development (2019) highlight the scale of this issue [101,102]. This raises concerns about whether enough attention is being paid to this problem. In recent years, little has been done to reduce water contamination by estrogens, and currently, there is no specific legislation regulating their presence and levels in water resources.

### 4.2. Water Quality Current Regulatory Frameworks

There is no doubt that water is an irreplaceable and invaluable resource because of its essential role in the life of organisms and its significance in various human activities [103]. The United Nations General Assembly acknowledged “The human right to water and sanitation” in General Assembly Resolution 64/292 (28 July 2010), and the Human Rights Council reaffirmed this in General Assembly Resolution 15/9 [104]. However, data from our review revealed the widespread presence of estrogens in water bodies worldwide. The European Commission and the U.S. Environmental Protection Agency (EPA) have implemented legislation to control contamination and ensure water quality [105,106].

In 2000, the European Union adopted the Water Framework Directive (WFD) to establish regulations aimed at improving water quality for Europe’s rivers, lakes, and groundwater by reducing and removing contamination and restoring its quality. The WFD includes a list of priority substances to be monitored in surface waters, with standards set by the Environmental Quality Standards Directive [105]. This list must be reviewed and updated every 6 years. On 20 March 2015, the European Commission established a watch list of substances, including estradiol, estrone, and ethynylestradiol, for Union-wide monitoring, with updates every two years. However, the monitoring obligation for these estrogens ceased in 2019 [103,107].

In the USA, the EPA regulates drinking water quality under the Safe Drinking Water Act, setting primary standards for over 90 contaminants [106]. However, no primary standards for estrogen have been established. The EPA’s Contaminant Candidate List (CCL) [108] identifies potential drinking water contaminants not yet subjected to national regulation, and only 17-alpha ethynyl estradiol is listed among estrogen contaminants provided in CCL 5 [109]. The CCL is periodically reviewed and updated when new data emerges.

There has been some progress in public policies regulating water quality, but it is still needed, as the data from this review revealed the global occurrence of estrogens in water resources. More active measures are essential to monitor and control the occurrence of estrogens in water and to develop effective strategies to combat this contamination [110,111]. Although removing these compounds from water can be costly, failing to do so may result in even higher costs. In line with achieving the Sustainable Development Goals [112], especially SDG 2: Zero Hunger and SDG 6: Clean Water and Sanitation, estrogens in water resources could disrupt ecosystems, reduce biodiversity, and contaminate food sources such as fish. This, in turn, impacts food security.

### 4.3. Current Environmental Monitoring Challenges of Estrogens

Despite the data shown here, the current review study also identified the following research gaps that need further exploration and development to improve environmental monitoring:

- Lack of standardized methods for the detection and quantification of estrogens in water resources.
- Several estrogens are present at trace levels (ng/L to µg/L) in water resources, requiring highly sensitive and selective analytical instruments.
- Complex environmental matrices can interfere with detection, requiring extensive sample preparation.
- Matrix effects and instrument limitations can lead to errors (false positives/negatives) in the detection and quantification of estrogens in different water resources.
- Lack of data on the interactions of estrogens with traditional and emerging contaminants and their effects on human, animal, and environmental health.

## 5. A One Health Approach

**Figure 5:**
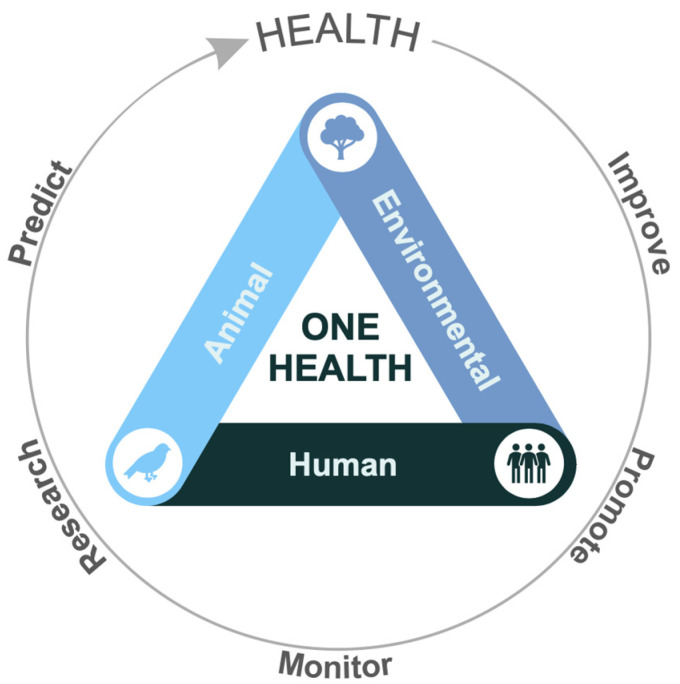
**The One Health approach.** The One Health recognizes the integration of the three areas of health (animal, environmental, and human) and understands that the promotion and maintenance of global health is achieved when integrated and collaborative measures occur in these areas together.

The current review showed the widespread global contamination of water resources with estrogens, and the One Health framework recognizes that environmental health, animal health, and human health are inextricably linked and must be addressed collectively (Figure 5). To improve global health and to address health challenges more effectively and sustainably, the One Health framework encourages joint strategies, such as the promotion and implementation of programs, policies, legislation, research, and monitoring of health data [113].

Studies have already demonstrated that environmentally relevant concentrations of estrogens induce ecotoxicity, particularly through endocrine disruption, resulting in reproductive deficiencies [114], developmental abnormalities [115], and immunological impairments [116]. Furthermore, within the environmental context, numerous other emerging contaminants have also been documented, such as pharmaceuticals [117], personal care products [118], pesticides [119], microplastics [120], and heavy metals [121]. However, the interactions between estrogens and these co-occurring contaminants remain poorly understood, as do the ecological risks posed by their mixtures. These interactions may produce synergistic, additive, or antagonistic effects, complicating risk assessment and environmental management strategies. Considering the One Health Framework, understanding the environmental occurrence of estrogens, as well as these complex mixtures, becomes essential for the development of integrative and effective mitigation strategies.

For the One Health paradigm, estrogens in water resources represent a growing and very complex challenge. From an environmental perspective, estrogens can cause endocrine effects in fish, reptiles, and amphibians. Feminization of males [122], infertility [123], malformations [124], and behavioral changes [125] have already been reported. Such effects can threaten species proliferation, directly harm biodiversity, and destabilize entire ecosystems. This issue becomes even more serious when considering food chains and interconnected ecosystems [126]. From an animal health perspective, estrogens in water resources can cause direct harm to domestic animals and livestock. Studies have shown hormonal imbalances [58,59], reproductive issues [127], and developmental changes [128]. Consequently, this poses direct economic challenges for livestock farming and indirect risks to human health through the consumption of contaminated animal products [58]. Lastly, in terms of human health, the main concern revolves around continuous and cumulative exposure to estrogens in contaminated water. Contact with these substances can occur through various pathways, such as direct ingestion of contaminated drinking water, consuming food grown with contaminated irrigation water, or skin contact while bathing in polluted water. Although our data indicates that estrogen levels in water resources are generally quite low, typically in the ng/L range, the potential effects of long-term exposure remain unknown. This uncertainty is even greater for vulnerable groups like children, the elderly, and individuals with hormonal disorders.

Given this scenario, the One Health approach recognizes that water pollution by estrogens is an environmental, animal, and human health issue [129]. Addressing this challenge and reducing the problem requires more than just environmental efforts; it needs interdisciplinary and cross-sector strategies involving researchers, professionals, and leaders in public health, veterinary medicine, environmental sciences, and health management. This reinforces the core idea of One Health: everyone’s health is interconnected [130]. These efforts should include basic actions like public education on environmental and health issues, especially the proper use and disposal of pharmaceuticals, systematic water quality monitoring, and promoting environmental awareness. At the same time, more advanced and resource-heavy measures are crucial, such as investment in research, developing improved wastewater treatment technologies, and implementing evidence-based public policies aimed specifically at regulating and controlling the presence of estrogens in water environments [131,132].

## 6. Conclusions

The revised data in this review showed that 39 estrogens were found in 40 water bodies across 59 countries, highlighting the widespread and global contamination of water resources by estrogens and their clear human-made origins. Alarmingly, estrogens were often detected in the effluents of water treatment plants, pointing to the inefficiency of current technologies in removing these compounds from water. Therefore, it is crucial to develop and implement more effective and targeted water treatment methods, along with updated regulations and monitoring systems that account for the complexity of estrogens.

From a One Health perspective, water contamination by estrogens poses a universal health threat. These compounds can disrupt the endocrine system, affect reproductive health in wildlife, alter aquatic ecosystems, and pose long-term risks to human health. All these interconnected impacts emphasize the need for an integrated, multi-sectoral approach that includes environmental, veterinary, and public health considerations. Only through a coordinated One Health strategy can we reduce the risks these substances pose and protect the health of ecosystems, animals, and humans alike.

## Figures and Tables

**Table 1:** Minimum and maximum concentration (ng/L) of each estrogen detected in water resources worldwide.

Estrogenic Compound	Min.	Max.
alfatradiol (17α-estradiol)	0.026	1250.0
diClE2	0.041	17.0
estradiol	0.002	7,988,120.0
estradiol-glucoside	105.5	105.5
estradiol-3-glucuronide	47.0	210.0
estradiol-3-sulfate	0.041	141.0
estradiol-3-β-D-glucuronide	1.8	1.8
estradiol-17-acetate	1.1	176.0
estradiol-17-glucuronide	1.1	7.34
estradiol-17-sulfate	79.8	79.8
estradiol-17-valerate	2.3	8.47
estradiol-17-β-D-glucuronide	0.4	1.8
estriol	0.003	83,430.0
estriol-3-glucuronide	37.0	150.0
estriol-3-sulfate	0.100	160.0
estriol-3-β-D-glucuronide	2.7	2.7
estriol-16-glucuronide	0.34	2.67
estrone	0.03	10,380,000.0
estrone-3-glucuronide	3.0	40.0
estrone-3-sulfate	0.041	255.0
estrone-3-β-D-glucuronide	0.2	17.0
ethinylestradiol	0.002	624,300.0
ethinylestradiol-3-glucuronide	0.23	5.85
ethinylestradiol-3-sulfate	0.080	178.0
E2-diS	160.0	1500.0
E2-monoS	3.3	6.6
E2-S&G	3.7	17.0
mestranol	1.94	110.0
monoBrEE2	0.041	0.041
2-bromo-17β-estradiol	21.0	36.0
2-hydroxyestrone	3.9	6.6
4-hydroxyestrone	6.3	10.3
2,4-dibromo-17β-estradiol	7.6	7.6
2,4-dichloro-17β-estradiol	15.0	18.0
4-chloro-estriol	<LOQ	<LOQ
4-chloro-17α-ethynylestradiol	4.2	4.2
16α-hydroxyestrone	2.4	51.7
16-ketoestradio	5.1	16.6
17α-estradiol-3-sulfate	170.0	170.0

<LOQ: below the limit of quantification.

**Table 2:** Minimum and maximum concentration (ng/L) of estrogens detected in water resources worldwide by water body.

Water Body	Min.	Max.
Bay water	1.62	20.9
Canal	0.018	10,380,000.0
Creek	0.125	4240.0
Coastal water	0.10	278.4
Dam water	0.004	7.0
Drainage extension	284,220.0	1,065,000.0
Drinking water	0.002	1,878,140.0
Effluent of domestic wastewater treatment plants	2.1	47.0
Effluent of fish farm	2.25	3.6
Effluent of livestock wastewater treatment plants	1.5	10,000.0
Embayment	1.58	1.87
Effluent of industrial wastewater treatment plants	1.8	120.0
Effluent of sewage treatment plants	0.04	102,500.0
Estuarine	0.04	176.0
Effluent of wastewater treatment plants	0.02	83,430.0
Groundwater	0.11	1745.0
Lake	0.03	925,240.0
Lagoon	0.58	5,142,900.0
Ocean	0.16	0.5
Pond	1.2	25.0
Reclaimed water	1.1	18.1
Reservoir	0.01	15.0
Residual water	270.0	400.0
River	0.002	7,988,120.0
Sea canal	0.5	0.5
Seawater	0.041	9.2
Spring water	0.01	0.17
Storm water runoff	2.3	240.0
Surface water	0.01	624,300.0
Tap water	0.1	9570.0
Tributary	0.5	12.0
Treated water	1.0	1.5
Unidentified water	0.75	1.68
Untreated wastewater discharged into surface waters	5.9	640.0
Waterway	0.2	0.6
Well water	0.1	47,310.0
Wetland water	0.3	103.0
Stream	0.04	556,340.0
Swimming pool water	4.0	43.0

## Data Availability

Supporting data is available in Open Science Framework (https://osf.io/rkvtf/ (accessed on 27 July 2025)).

## Supplementary Materials

The following supporting information can be downloaded at: https://www.mdpi.com/article/10.3390/jox15050148/s1. Supplementary Material S1: Search strategy. Supplementary Material S2: Concentration (ng/L) of estrogens detected in water samples from different sources worldwide.
