# Supplementary material for: A Systematic Review of Estrogens as Emerging Contaminants in Water: A Global Overview Study from the One Health Perspective
Source: J Xenobiot. 2025 Sep 13;15(5):148. doi: 10.3390/jox15050148 (PMC12452746; doi:10.3390/jox15050148)
Supplement: Supplementary file 1 [file jox-15-00148-s001.zip › Supplementary material 1.pdf]

SUPPLEMENTARY MATERIAL FILE 1. Search strategy. The complete search strategy syntax for PubMed, Scopus, and Web of Science databases.

**DATABASE**	**PubMed**
Limits	Date range: all years
Search date	October 10th, 2022, at 11:10 a.m.
Works found	2,134
Search query	(estradiol OR estrone OR estriol OR ethinylestradiol OR mestranol OR quinestrol OR hydroxyestrones OR "ethynodiol diacetate" OR "esterified estrogens" OR "conjugated estrogens" OR "catechol estrogens") AND ("water resources" OR "drinking water" OR effluent OR "surface freshwater" OR wastewater OR "residual water" OR "water body" OR "tap water")

**DATABASE**	**Scopus**
Limits	Date range: all years
Search date	October 10th, 2022, at 11:32 a.m.
Works found	3,028
Search query	(TITLE-ABS-KEY (estradiol) OR TITLE-ABS-KEY (estrone) OR TITLE-ABS-KEY (estriol) OR TITLE-ABS-KEY (ethinylestradiol) OR TITLE-ABS-KEY (mestranol) OR TITLE-ABS-KEY (quinestrol) OR TITLE-ABS-KEY (hydroxyestrones) OR TITLE-ABS-KEY ("ethynodiol diacetate") OR TITLE-ABS-KEY ("esterified estrogens") OR TITLE-ABS-KEY ("conjugated estrogens") OR TITLE-ABS-KEY ("catechol estrogens") AND TITLE-ABS-KEY ("water resources") OR TITLE-ABS-KEY ("drinking water") OR TITLE-ABS-KEY (effluent) OR TITLE-ABS-KEY ("surface freshwater") OR TITLE-ABS-KEY (wastewater) OR TITLE-ABS-KEY ("residual water") OR TITLE-ABS-KEY ("water body") OR TITLE-ABS-KEY ("tap water"))

**DATABASE**	**Web of Science**
Limits	Date range: all years
Search date	October 10th, 2022, at 11:45 a.m.
Works found	2,694
Search query	(ALL=(estradiol OR estrone OR estriol OR ethinylestradiol OR mestranol OR quinestrol OR hydroxyestrones OR "ethynodiol diacetate" OR "esterified estrogens" OR "conjugated estrogens" OR "catechol estrogens")) AND ALL=("water resources" OR "drinking water" OR effluent OR "surface freshwater" OR wastewater OR "residual water" OR "water body" OR "tap water")
